# Development and characterization of a reverse genetics system for the lineage II Chicava strain of Machupo virus in a guinea pig model

**DOI:** 10.1371/journal.pntd.0012834

**Published:** 2025-01-24

**Authors:** Emily Mantlo, Junki Maruyama, John T. Manning, Rachel A. Reyna, Cheng Huang, Slobodan Paessler

**Affiliations:** 1 Department of Microbiology and Immunology, University of Texas Medical Branch, Galveston, Texas, United States of America; 2 Department of Pathology, University of Texas Medical Branch, Galveston, Texas, United States of America; Beijing Children’s Hospital Capital Medical University, CHINA

## Abstract

**Background:**

Machupo virus (MACV) is a New World mammarenavirus (hereafter referred to as “arenavirus”) and the etiologic agent of Bolivian hemorrhagic fever (BHF). No vaccine or antiviral therapy exists for BHF, which causes up to 35% mortality in humans. New World arenaviruses evolve separately in different locations. To date, up to eight lineages of MACV have been identified in Bolivia. While the prototype MACV Carvallo strain belongs to lineage I discovered in the Memore Province in the 1960s, the MACV lineage II strains have become the dominantly-circulating lineage in the same province since 1993.

**Methods:**

We report the development of a reverse genetics system for the MACV lineage II Chicava strain, using a pRF42 plasmid encoding the L and S segment genomic RNA under the transcriptional control of a murine DNA-dependent RNA polymerase I promoter sequence. Rescue of the recombinant MACV Chicava strain (rMACV-Chicava) was accomplished by expression of the L protein and nucleoprotein genes of the MACV Carvallo strain *in trans* in transfected baby hamster kidney (BHK-21) cells. We characterized the multiplication kinetics of rMACV-Chicava in African green monkey kidney epithelial Vero cells, followed by determining the virulence phenotype in outbred Hartley guinea pigs.

**Principal findings:**

We demonstrated that the multiplication kinetics in Vero cells, virulence phenotype in guinea pigs, and neutralizing antibody titers are indistinguishable between rMACV-Chicava and the wild-type parental virus.

**Conclusion and significance:**

We conclude that rMACV-Chicava provides a useful model system to investigate the emergence of MACV lineage II strains and the guinea pig model has utility for the development of candidate vaccines and therapeutic antibodies for BHF.

## Author summary

Machupo virus (MACV) is a New World arenavirus that causes Bolivian hemorrhagic fever (BHF) in humans. BHF involves both viscerotropic and neurotropic disease. The prototypical Carvallo strain frequently used for MACV pathogenesis and vaccine development studies has been proven to be fully or partially attenuated in immunocompetent mice and guinea pigs. To overcome this challenge, we report the development of a reverse genetics system for the MACV Chicava strain that is representative of the dominantly-circulating lineage in the Mamore province. Our reverse genetics system rescues the recombinant MACV Chicava strain from BHK-21 cells transfected with Pol-I-driven plasmids transcribing viral genomic RNAs and expression plasmids of the L protein and nucleoprotein genes. The recombinant MACV Chicava strain exhibits the virulence phenotype and antigenicity similar to the parental wild-type virus. The lethal guinea pig model developed in this study showed both viscerotropic and neurotropic disease and, hence, has broad utility for molecular virology research and the development of candidate vaccines and therapeutic antibodies for human BHF.

## Introduction

Machupo virus (MACV) is the causative agent of Bolivian hemorrhagic fever (BHF), a neglected tropical disease. As a member of the genus *Mammarenavirus* within the family *Arenaviridae*, MACV is evolutionarily related to other pathogenic arenaviruses, e.g., Lassa virus (LASV) and Junín virus (JUNV) [1]. Mammarenaviruses have a bi-segmented genome consisting of two genomic segments that each encodes two viral genes using an ambisense coding strategy [2]. The small (S) segment encodes the glycoprotein complex (GPC) and the nucleoprotein (NP), while the large (L) segment encodes the RNA-dependent RNA polymerase (L) and the small matrix protein (Z)[3–5]. BHF has a biphasic disease pattern consisting of an initial non-specific prodromal phase followed by severe hemorrhagic/neurologic symptoms. The overall case-fatality rate of BHF ranges from 20–35% and no antiviral therapy or vaccine is available for MACV [6,7]. Therefore, further research is needed to understand MACV pathogenesis and develop appropriate countermeasures.

The geographic distribution of MACV is correlated with the presence of its reservoir species, the vesper mouse (*Calomys callosus*). Accordingly, BHF is endemic within the Beni District of northern Bolivia [6], where humans exposed to rodents, such as agricultural workers, are at the highest risk of developing BHF. Geography is an important factor shaping the evolution of MACV. There are eight lineages of MACV (I, II, III, IV, V, VI, VII, and VIII) identified to date [8]. Most isolates belong to lineages I, II, V, and VII and are localized to two provinces within the Beni district. Lineages I and II are comprised of isolates collected in the Mamore province, while lineages V and VII consist of isolates from the Itenez province [8,9]. The prototypical Carvallo strain was isolated from a fatal human case in the Mamore province in 1963 [9,10], and there were no cases of BHF reported between 1974–1992. Since 1993, the re-emergence of MACV in the Mamore province has sparked several outbreaks. These more recent isolates belong to lineage II, which is distinct from the Carvallo strain and other lineage I strains collected in the 1960s [8,9]. Similarly, recent isolates collected in the Itenez province between 2000 and 2008 belong to lineage V and differ from lineage VII strains detected prior to 1995. It has therefore been speculated that lineage II and V viruses may have replaced lineage I and VII viruses in their respective geographical regions [9]. Furthermore, lineage II and V viruses are associated with the most recent 2007–2008 re-emergence of MACV [9]. However, the molecular basis of either displacement event is poorly understood, warranting the development of reverse genetics systems for the dominantly circulating lineages of MACV to identify the viral encoded determinants for virulence.

We report here the development of a reverse genetics system for the lineage II Chicava strain of MACV, using the same plasmid backbones originally incorporated in the reverse genetics system of the prototype Carvallo strain [11]. The Carvallo and Chicava strains differ by 2.9% and 3.9% in nucleotide sequence in the S and L segments, respectively. Interestingly, the Carvallo strain is uniformly lethal in immunocompromised STAT-1^-/-^ mice and IFN-αβ/γR^-/-^ mice [12,13] but is partially attenuated in the outbred Hartley guinea pig model, presumably due to the extensive but poorly defined passage history of the Carvallo strain in cell culture [14,15]. By contrast, the Chicava strain has been demonstrated to cause uniformly lethal disease in guinea pigs even at low doses [16]. In addition, the Chicava strain of MACV produces a biphasic disease reminiscent of that observed in humans. In guinea pigs, clinical signs appear around one week post-inoculation and include weight loss, fever, skin erythema, dyspnea, and bloody diarrhea. Neurologic signs present at 16–20 days post-exposure and include head tilt, ataxia, and hind limb paralysis [14]. These animal studies demonstrate the utility of the Chicava strain of MACV in consistently replicating lethal human BHF. While one other group has recently rescued a 2000 isolate of MACV belonging to lineage V [17], no lineage II MACV strain has thus far been rescued using reverse genetics. Herein, we demonstrate that the recombinant Chicava strain of MACV rescued using the reverse genetics system resembles the virulence phenotype of the original stock virus, suggesting that this reverse genetics system can also support the identification of virulence determinants underlying the switch of the dominantly circulating lineage in the Mamore province.

## Materials and methods

### Cells, viruses, and biosafety

Vero cells (ATCC CCL-81) and BHK-21 cells (ATCC CCL-10) were maintained in Dulbecco’s modified Eagle’s medium (DMEM) (Gibco) supplemented with 10% fetal bovine serum (FBS, Gibco) and 1% penicillin/streptomycin (P/S). The wild-type Carvallo strain of MACV (Genbank accession no. JN794583.1 and JN794584.1) and the Chicava strain of MACV (Genbank accession no. AY624355.1 and AY624354.1) were obtained from Dr. Thomas G. Ksiazek. Wild-type and recombinant viruses were propagated by inoculating Vero cells with virus at a multiplicity of infection (MOI) of 0.01. Supernatant was collected at 72–96 hours post-infection (hpi), briefly centrifuged to remove cell debris, and then added to Amicon Ultra 100K Filter Devices for centrifugation to further purify and concentrate virus. All work with infectious MACV was performed at the Galveston National Laboratory (GNL) biosafety level-four (BSL-4) facilities, following institutional biosafety protocols at the University of Texas Medical Branch and guidelines of the Federal Select Agent Program.

### Generation of MACV (Chicava strain) plasmids

Vero cells were infected with MACV (Chicava) and lysed at 96 hpi in Trizol reagent. Total RNA was isolated using the DirectZol RNA microplus kit (Zymo Research) and viral cDNA was reverse-transcribed by SuperScript III Reverse Transcriptase using gene specific primers (ThermoFisher). Following PCR amplification of the full-length viral genome, DNA segments were separated by agarose gel electrophoresis, gel purified, trimmed by restriction endonucleases recognizing naturally occurring cutting sites in the viral genome, and cloned into the murine Pol-I-driven pRF42 plasmid in antigenomic orientation. Both plasmids were fully sequenced and confirmed to contain the expected sequences.

The full bi-segmented genome of the MACV Chicava strain (Genbank Accession No. AY624355.1 and AY624354.1) was sequenced and inserted into the murine Pol-I driven pRF42 plasmid previously used for the reverse genetics system of MACV Carvallo strain [11]. Briefly, the 5’ and 3’ ends of MACV genomic segments each contain an endogenous *AvrII* restriction site for the insertion of cDNA encoding the S and L segments into the pRF42 plasmid using previously published methods [11], to create pRF42-MACV-Chicava-S and pRF42-MACV-Chicava-L. The engineering of the synonymous A525G nucleotide substitution in the L segment provided a marker for the recombinant MACV Chicava (rMACV-Chicava).

### Rescue of rMACV

**Fig 1:**
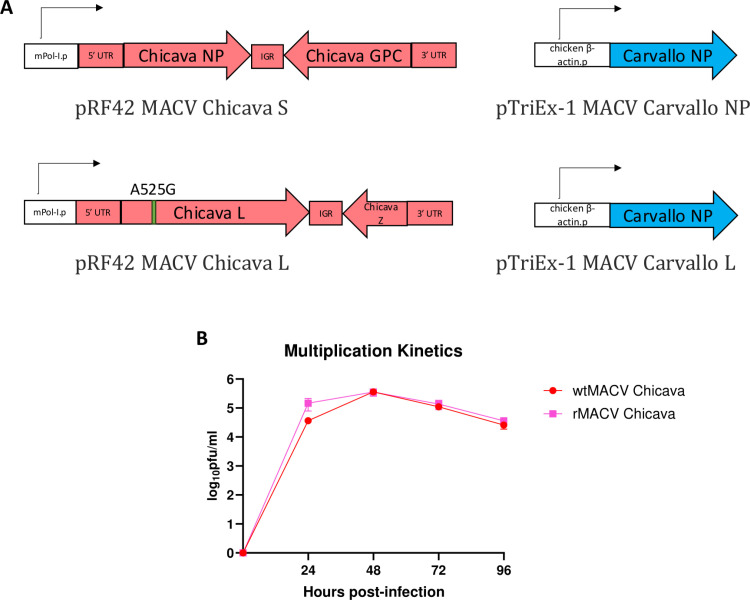
Rescue of MACV Chicava. **(A)** Schematic of plasmids used for rescue of rCHICV. Two plasmids containing the full-length MACV Chicava S and L segments including the 5’ and 3’ untranslated regions (UTR) and intergenic regions (IGR) downstream of a murine Pol-I promoter (mPol-I.p) were transfected alongside two expression plasmids encoding the MACV Carvallo NP and L genes downstream of a chicken β-actin promoter (chicken β-actin.p). The silent A525G single-nucleotide substitution was added in the L segment as a marker for recombinant virus. **(B)** Multiplication kinetics of rescued MACV Chicava compared to the wild-type isolate in Vero cells at a MOI of 0.01. The mean of three replicates is shown, with error bars displaying standard deviation.

Rescue of rMACV-Chicava was undertaken by co-transfection of four plasmids into BHK-21 cells, including pRF42-MACV-Chicava-L, pRF42-MACV-Chicava-S, pTriEx-1-MACV-Carvallo-NP, and pTriEx-1-MACV-Carvallo-L plasmids [18](Fig 1A). An equimolar concentration of the two plasmids encoding full-length S and L sequences from MACV (Chicava) as well as two chicken β-actin promoter-driven pTriEx-1 expression plasmids containing the MACV Carvallo NP and L protein sequences were co-transfected using the Xtreme Gene9 transfection reagent. These expression plasmids had originally been constructed for rescue of the Carvallo strain of MACV, as previously described [11]. Supernatant was collected at 96 hours post-transfection and passaged once on Vero cells. At 5–7 dpi, when cytopathic effect was observed, p1 viruses were collected. To determine the sequence of rescued virus, cDNAs were amplified via RT-PCR, purified using a QIAquick PCR purification kit (Qiagen) and sequenced using an ABI Prism 3130xl DNA sequencer (Life Technologies).

### Virus multiplication kinetics

Vero cells were infected in triplicate with wild-type or recombinant stocks of the Chicava strains of MACV at a multiplicity of infection (MOI) of 0.01. Supernatants were harvested every 24 hours for up to four days. Virus titrations were performed by plaque assay as previously described [19].

### Animal studies

Six-to-eight week old outbred Hartley guinea pigs (Charles River Laboratories) were inoculated with 10^4^ plaque-forming units (PFU) of wild-type or recombinant stocks of either the Carvallo or Chicava strains of MACV via the intraperitoneal (IP) route. Clinical signs and weights were recorded daily for the first 24 days of the acute infection. One week before challenge, temperature-sensitive transponders were implanted into each guinea pig for the monitoring of body temperatures, and measurements were recorded daily following challenge. Animals that developed more than 20% weight loss, hypothermia (defined as body temperature lower than 33°C), or neurological signs of disease were humanely euthanized. All animal work was reviewed and approved by University of Texas Medical Branch Institutional Animal Care of Use Committee (protocol number #1904038).

### 50% plaque reduction neutralization test (PRNT_50_)

Blood was collected from all animals at time of euthanasia or at the end of the study at 30 dpi. Sera was purified from whole blood and heat-inactivated at 56°C for 30 min. Serum samples were then serially diluted from 1:15 to 1:480, in two-fold increments, and mixed with an equal volume of 80 PFU of MACV. The serum-virus mixture was incubated at 37°C for one hour, inoculated into 12-well plates of Vero cells, and adsorbed at 37°C for one hour before addition of tragacanth overlay (1.2% tragacanth gum mixed with equal volume of Temin’s 2X MEM containing 4% FBS and 2% P/S). Plates were incubated for 7–8 days before fixation and staining with 0.25% crystal violet in 10% formalin. The PRNT_50_ titer was determined based on the lowest dilution, at which plaque number is reduced by 50% or greater in comparison with the virus control wells.

### Histopathology

Upon necropsy, brains, lungs, livers, spleens, and kidneys were collected in 10% buffered formalin for histopathological analysis. Samples were fixed for a minimum of 5 days before transfer to 70% ethanol. Tissues were then trimmed, paraffin-embedded, sectioned into 5 μm slices, and stained with hematoxylin and eosin (H&E) as previously described [19].

### Statistical analysis

Because viral growth curve data did not follow normal distribution, a Wilcoxon matched-pairs signed rank test was used to compare the multiplication kinetics between the wild-type and recombinant viruses. A Kaplan-Meier survival curve was generated for the animal study. All statistical analyses were performed via GraphPad Prism software.

## Results

### Rescue of MACV Chicava strain

Expression of NP and L proteins *in trans* is required for the rescue of recombinant MACV [20,21]. Importantly, the close evolutionary relationship between the Carvallo and Chicava strains results in a highly conserved amino acid (aa) sequence in the NP and L proteins (NP protein: 99.1% aa sequence homology; L protein 97.6% aa sequence homology) (S1 Table). We therefore expected that the NP and L proteins encoded in the Carvallo strain would facilitate the transcription of the S and L genomic segments of the Chicava strain and, consequently, generate infectious viruses. Alongside the newly-generated pRF42-MACV-Chicava-S and pRF42-MACV-Chicava-L containing the full-length genomic sequence of MACV Chicava, we used plasmids previously created to express the NP and L protein of MACV Carvallo strain, i.e., pTriEx-1-MACV-Carvallo-NP and pTriEx-1-MACV-Carvallo-L, in vertebrate cells. rMACV-Chicava was rescued via the co-transfection of BHK-21 cells with these four plasmids followed by a single passage in Vero cells. Total RNA was then extracted from the infected cells and amplified by RT-PCR. We detected the consensus A525G nucleotide substitution in the L segment, confirming the rescue of rMACV-Chicava.

We next compared the multiplication kinetics of rMACV-Chicava to the original stock of wild-type MACV Chicava strain (wtMACV-Chicava) in Vero cells. There was no demonstrable difference in the multiplication kinetics between rMACV-Chicava and wtMACV-Chicava from 0–96 hours post-infection (hpi). The highest titer of wtMACV-Chicava and rMACV-Chicava was both 5.5±0.1 log_10_PFU/mL at 48 hpi (*P* > 0.99) (Fig 1B). To summarize, expression of the L and NP protein of the Carvallo strain supports the rescue of rMACV-Chicava in transfected BHK-21 cells, and the resultant rMACV-Chicava exhibited no difference in multiplication kinetics from wtMACV-Chicava in cell culture.

### rMACV-Chicava resembles the virulence phenotype of wtMACV-Chicava in guinea pigs

**Fig 2:**
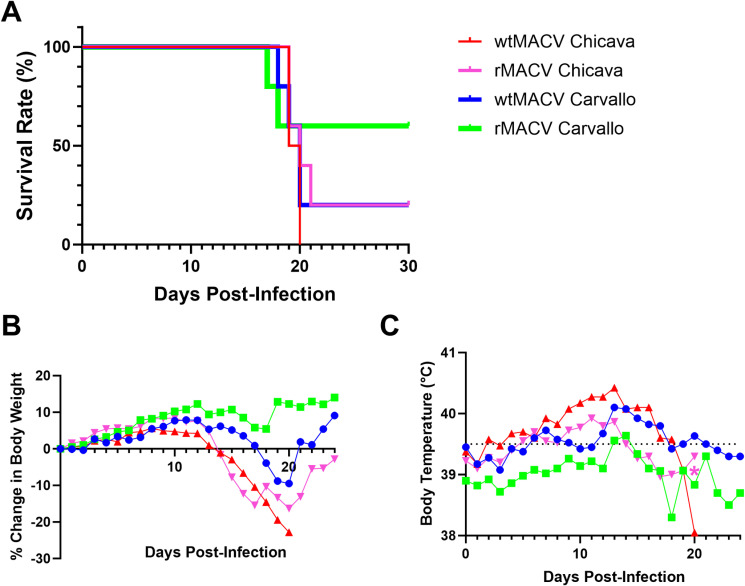
Wild-type and recombinant MACV produce similar disease in 6–8 week-old Hartley guinea pigs. **(A)** Survival rate of guinea pigs (n = 5 per group) challenged with 10^4^ pfu of the indicated MACV strains. **(B)** Mean body weight change compared to baseline weight. **(C)** Mean body temperature. The dotted line indicates the clinical definition of fever (39.5°C) for a 6–8-week-old guinea pig. Pink asterisk (*) indicates a broken transponder that prevented the collection of further body temperature readings in one surviving animal (#49).

To assess whether rMACV-Chicava exhibits a similar virulence phenotype to that of wtMACV-Chicava in the guinea pig model, two groups of five Hartley guinea pigs were each challenged via the IP route with 10^4^ PFU of rMACV-Chicava or wtMACV-Chicava. Two additional groups of five animals were infected with the recombinant MACV Carvallo strain rescued from the reverse genetics system and wild-type MACV Carvallo strain for comparison. As anticipated, wtMACV-Chicava strain produced uniform lethality (Fig 2A). Although one animal survived the challenge with rMACV-Chicava, there was no distinguishable difference in the Kaplan-Meier survival curve between rMACV Chicava and wtMACV Chicava. While the IP challenge with wtMACV Carvallo also resulted in 80% mortality (4/5), the mortality rate of animals infected with rMACV was lower (40%, 2/5) without reaching significant difference (*P* = 0.64). Clinical signs observed among animals in all four groups included scruffy fur and hunched posture. Infected guinea pigs in all four groups began to lose weight after 13 days post infection (dpi) (Fig 2B). Elevated temperatures were also recorded for multiple animals in all four groups (Fig 2C). In addition, hind limb paralysis, a sign of neurological disease, developed in animals infected with wtMACV Carvallo, rMACV Carvallo, and rMACV Chicava. All animals that succumbed to challenge with wtMACV Chicava and 3 out of 4 animals that succumbed to challenge with rMACV Chicava were humanely euthanized after losing 20% of their body weight and before developing neurological disease. Conversely, all animals that succumbed to the IP challenge with rMACV-Carvallo and wtMAC-Carvallo developed hind limb paralysis prior to euthanasia, while only one animal infected with rMACV-Chicava developed neurological disease. Taken together, both rMACV-Chicava and rMACV-Carvallo resembled the virulence phenotype of the respective parental wild-type viruses in guinea pigs.

**Fig 3:**
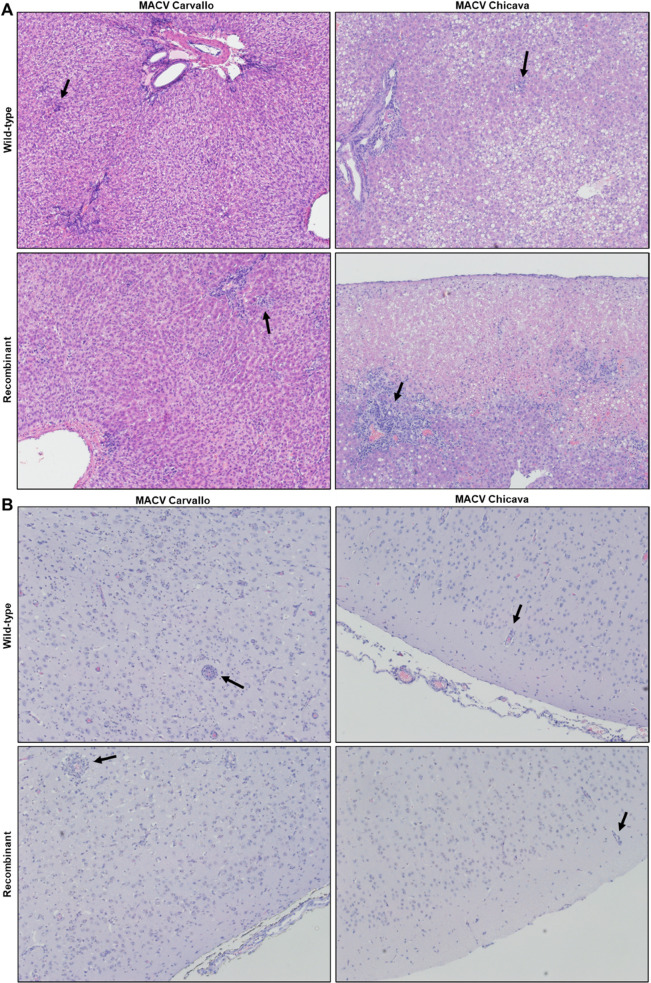
Wild-type and recombinant MACV cause inflammation in the liver and brain. Representative H&E-stained images of **(A)** liver and **(B)** brain tissues collected at the time of euthanasia. Images are representative of animals that succumbed to challenge. Black arrows indicate areas of **(A)** focal hepatic inflammation or **(B)** perivascular cuffs.

Because MACV is known to cause both viscerotropic (liver) and neurotropic disease in vertebrate hosts, we examined histopathological changes in the liver and brain of all infected guinea pigs. Pathological changes were consistent with the inflammatory viscerotropic and neurotropic disease that is characteristic of BHF. In the liver, inflammation was observed both in the periportal regions and in scattered foci (Fig 3A). Infection with either wtMACV or rMACV Chicava also produced fatty steatosis throughout the lobule. In the brain, extensive inflammatory cell infiltration and perivascular cuffing were observed (Fig 3B). No distinctive differences were noted between tissues infected with the wild-type vs. recombinant MACV Carvallo viruses, and similarly no differences were noted between tissues infected with wild-type vs. recombinant Chicava viruses.

### Infection with rMACV Chicava results in similar neutralizing antibody titers as infection with wtMACV Chicava

**Fig 4:**
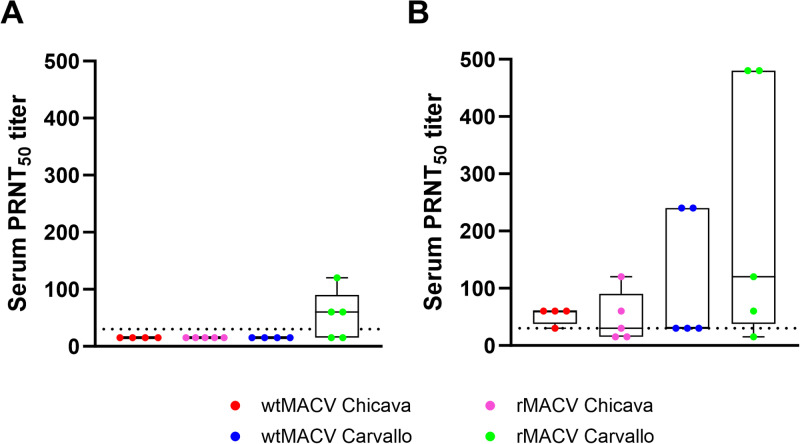
Guinea pigs infected with recombinant MACV Chicava produce a heterologous neutralizing antibody response. **(A)** Individual serum PRNT_50_ titers against wtMACV Carvallo or **(B)** rMACV Carvallo GPC_ΔN83/N166/F438I_. Box plots extend from the 25^th^ to 75^th^ percentiles, with the center line indicating the median. Whiskers represent the minimum and maximum values. The dotted line indicates the lower limit of detection (LOD), i.e., 1:30 PRNT_50_ titer. Samples with no neutralizing activity detected were plotted at 1:15 PRNT_50_ titer (50% LOD).

Most guinea pigs did not succumb to the IP challenge until 17–21 dpi. Therefore, serum samples were collected from animals either at the time of euthanasia or at the end of this study, i.e., 30 dpi. We undertook PRNT_50_ to compare the serum neutralizing activity elicited by rMACV-Chicava, wtMACV-Chicava, rMACV-Carvallo, and wtMACV-Carvallo. Initial PRNT_50_ was undertaken using wtMACV Carvallo. The three surviving animals challenged with rMACV Carvallo all developed neutralizing antibodies against wtMACV Carvallo. However, no serum neutralizing activity was detected in animals euthanized between 17 and 21 dpi. Additionally, no other surviving animals developed serum neutralizing activity greater than 1:30 PRNT_50_ (Fig 4 and S2 Table). The MACV GPC contains N-linked glycans that have been demonstrated to interfere with antibody-mediated neutralization [22]. Therefore, we determined PRNT_50_ titers against the MACV Carvallo GPC_ΔN83/N166/F438I_ mutant that lacks two N-linked glycans (rMACV Carvallo GPC_ΔN83/N166/F438I_) [23]. Significantly, most animals (84%, 16/19) showed serum neutralizing activity against rMACV Carvallo GPC_ΔN83/N166/F438I_ despite varying geometric mean PRNT_50_ titers between four groups (wt MACV-Carvallo: 68.9; rMACV-Carvallo: 120, wtMACV-Chicava: 50.5; rMACV-Chicava: 34.5). Importantly, there is no significant difference in either the prevalence of serum neutralizing antibodies or the geometric mean PRNT_50_ titer between wtMACV-Chicava and rMACV-Chicava (wtMACV-Chicava: 100%, 4/4; rMACV-Chicava: 60%, 3/5). The sole surviving animal of rMACV Chicava infection developed the highest titer (120) among all MACV Chicava-infected animals. This suggests that infection with either wtMACV-Chicava or rMACV-Chicava results in the production of similar levels of neutralizing antibodies over time.

## Discussion

In this study, we constructed the first reverse genetics system to rescue recombinant MACV lineage II Chicava strain. Expression of the L and NP proteins of the Carvallo strain *in trans* supported the rescue of rMACV-Chicava despite the aa sequence diversity. Our system proves that the L and NP proteins expressed *in trans* can be compatible for the transcription of the L and S segment of a heterologous lineage of MACV. We used the pRF42 plasmid that contains a murine DNA-dependent RNA polymerase I promoter to develop the reverse genetics system of the MACV Chicava strain, a different system from the T7 promoter-driven expression plasmids described by Jain and colleagues [17]. Further, the rescue of the Carvallo and Chicava strain warrants the evaluation of our system in rescuing the five other lineages of MACV. If successful, our techniques can support the investigation of the emergence of lineage II and lineage V strains that became the dominantly circulating lineages in the Mamore and Itenez Provinces, respectively. Specifically, comparative studies between rMACV-Carvallo and rMACV-Chicava chimeras in guinea pigs would potentially provide the fundamental knowledge of how MACV lineage II became the dominantly circulating genetic lineage in nature.

rMACV-Chicava demonstrated similar multiplication kinetics in cell culture and a similar virulence phenotype in guinea pigs to that of the parental wt virus, although our conclusions can only be limited to Vero cells and 6–8-week-old Hartley guinea pigs. The sole guinea pig that survived the IP challenge with rMACV-Chicava at 10^4^ PFU (animal #49) showed signs of infection, i.e., significant weight loss and fever, but recovered from acute disease prior to 30 dpi, the endpoint of the challenge experiment. Further, the same animal developed the highest serum neutralizing activity against rMACV Carvallo GPC_ΔN83/N166/F438I_ at 1:120 PRNT_50_. The most plausible explanation is that outbred Hartley guinea pigs may exhibit variation in the sensitivity to the lethal challenge of MACV. We also cannot discount the possibility that rMACV-Chicava acquired mutations that attenuated the virulence phenotype in the surviving animal, though we were unable to isolate viral RNA from this animal to confirm the viral sequence. Guinea pigs challenged with rMACV-Chicava and wtMACV-Chicava exhibited 80% and 100% mortality in this study, respectively. In contrast, the mortality rates caused by rMACV-Carvallo and wtMACV-Carvallo were 40% and 80%, respectively. Importantly, the prototype MACV Carvallo strain was originally isolated in 1962 and differs in passage history between laboratories, as evidenced by slight variation in reported GenBank sequences (accession numbers AY358021.2, JN794584.1, AY216511.2). Different passage history resulted in variations in the available Genbank sequences of the MACV Carvallo strain, reflecting the partially attenuated phenotype observed in immunocompetent animals [14]. Therefore, we speculate that rMACV-Chicava will be better suited for identifying candidate vaccines and antiviral therapies. Identification of attenuation determinants accumulated during the passage of the Carvallo strain will potentially generate knowledge to support the rational design of candidate live-attenuated vaccines for BHF. Additionally, because rMACV-Chicava exhibits the virulence phenotype in guinea pigs, our system provides a valuable tool to examine the attenuating effect of each mutation.

Some minor differences in the virulence phenotype of the Carvallo and Chicava strains were noted despite the small sample size. Liver pathology between animals challenged with wtMACV-Chicava/rMACV-Chicava was noticeably different from animals challenged with wtMACV-Carvallo/rMACV-Carvallo, with widespread fatty steatosis only observed in animals infected with either Chicava strain and noticeably absent in animals challenged with either Carvallo strain. All but one animal infected with either Chicava strain developed rapid severe weight loss that necessitated euthanasia, and this rapid weight loss likely contributed to the severe fatty steatosis observed in the livers of animals challenged with either rMACV-Chicava or wtMACV-Chicava [24]. Only one animal challenged with rMACV-Chicava developed neurological disease, namely hind limb paralysis, whereas all animals that succumbed to challenge with rMACV Carvallo or wtMACV Carvallo developed hind limb paralysis. This is consistent with one previous study that reported the appearance of hind limb paralysis in guinea pigs infected with the Carvallo strain, but not the Chicava strain, supporting our conclusion that rMACV-Chicava resembles the virulence phenotype of wtMACV-Chicava [14]. Since the onset of neurological disease is often late in the course of infection [16,25], we hypothesize that the high mortality rate in guinea pigs challenged with the Chicava strain precludes further progression to the late-stage neuroinvasive disease and a different dosage of IP challenge that does not trigger the rapid onset of mortality may be necessary for the development of a neuroinvasive disease guinea pig model using rMACV-Chicava [16]. Development of animal models that capture the viscerotropism and neurotropism of MACV will be helpful for the evaluation of candidate vaccines and antiviral therapies for BHF. Therefore, the rMACV Chicava strain generated in this study can be used in the guinea pig model to accurately mimic the acute viscerotropic and neurotropic disease observed in human cases of BHF, allowing for the study of MACV pathogenesis in both the visceral organs and brain and the evaluation of candidate vaccines and therapeutics.

Importantly, both wtMACV-Chicava and rMACV-Chicava elicit neutralizing antibody responses reactive with the heterologous MACV Carvallo strain. Antibodies raised against arenaviruses are often highly specific to one species and can even be strain-specific [26,27]. The cross-reactive antibodies recognizing both the Carvallo and Chicava strains warrant further analysis of epitopes conserved between different lineages of MACV to generate information relevant to the immunogenicity of candidate BHF vaccines. As expected, animals infected with rMACV Chicava produce similar neutralizing antibody titers as animals infected with wtMACV Chicava. We observed that the MACV Carvallo GPC_ΔN83/N166/F438I_ mutant is more sensitive to antibody-mediated neutralization than wtMACV-Carvallo, reflecting the arenavirus-conserved immune-escaping mechanism called “glycan shielding” [22,28–30]. This demonstrates that antisera collected from infected guinea pigs contains antibodies targeting neutralizing epitopes normally masked by glycans. Investigation of protection offered by these anti-MACV antibodies may provide strategies for the development of candidate therapeutic antibodies against BHF. Studies have shown that neutralizing epitopes hindered by the glycan shield of human immunodeficiency virus-1 can be recognized by monoclonal antibodies containing ultralong variable regions [31]. We speculate a similar strategy may be effective for the development of candidate therapeutic antibodies against MACV and, potentially, other arenaviruses.

Overall, the results of this study demonstrate the utility of the MACV Chicava reverse genetics system for generating recombinant viruses that are phenotypically indistinguishable from the wt parental virus. The engineering of mutations using a more recent lineage II isolate of MACV may provide a greater insight into the pathogenesis of MACV strains that have the greatest potential for re-emergence [9]. Furthermore, the Chicava reverse genetics system can uniquely be used to investigate the molecular determinants for the evolutionary displacement of lineage I MACV strains by lineage II strains. A greater understanding of the evolution of MACV within the endemic foci can lead to genetic surveillance of circulating MACV strains to predict and combat re-emergence in the human population.

## Supporting information

S1 Data **Fig 1a Raw Data.** Multiplication kinetics of rescued MACV Chicava compared to the wild-type isolate in Vero cells at a MOI of 0.01. Values represent PFU/mL. **Fig 2a Raw Data.** Survival of guinea pigs (n = 5 per group) challenged with 10^4^ pfu of the indicated MACV strains. A = alive, EU = euthanized. Percentages indicate survival rate of animals in each group at each dpi. **Fig 2b Raw Data.** Body weight loss by percentage as compared to baseline (0 dpi). **Fig 2c Raw Data.** Body temperature readings of infected guinea pigs. A red asterisk (*) indicates a broken transponder chip that prevented collection of further body temperature readings.
(XLSX)

S1 Table Amino acid differences in the NP and L protein between the Carvallo and Chicava strains. (DOCX)

S2 Table Serum PRNT50 titers for animals challenged with respective MACV strains. (DOCX)

## References

[1] Maes P, Adkins S, Alkhovsky SV, Avšič-Županc T, Ballinger MJ, Bente DA, et al. Taxonomy of the order Bunyavirales: second update 2018. Arch Virol. 2019;164(3):927–41. doi: 10.1007/s00705-018-04127-3 ; PMCID: PMC6581445 30663021 PMC6581445

[2] Radoshitzky SR, Buchmeier MJ, Charrel RN, Clegg JCS, Gonzalez J-PJ, Günther S, et al. ICTV virus taxonomy profile: Arenaviridae. J Gen Virol. 2019;100(8):1200–1. doi: 10.1099/jgv.0.001280 31192784 PMC12139605

[3] Poch O, Sauvaget I, Delarue M, Tordo N. Identification of four conserved motifs among the RNA-dependent polymerase encoding elements. EMBO J. 1989;8(12):3867–74. doi: 10.1002/j.1460-2075.1989.tb08565.x 2555175 PMC402075

[4] Salvato M, Shimomaye E, Oldstone MB. The primary structure of the lymphocytic choriomeningitis virus L gene encodes a putative RNA polymerase. Virology. 1989;169(2):377–84. doi: 10.1016/0042-6822(89)90163-3 2705303

[5] Perez M, Craven RC, de la Torre JC. The small RING finger protein Z drives arenavirus budding: implications for antiviral strategies. Proc Natl Acad Sci U S A. 2003;100(22):12978–83. Epub 2003/10/16. doi: 10.1073/pnas.2133782100 ; PMCID: PMC240730 14563923 PMC240730

[6] Patterson M, Grant A, Paessler S. Epidemiology and pathogenesis of Bolivian hemorrhagic fever. Curr Opin Virol. 2014;5:82–90. Epub 2014/03/15. doi: 10.1016/j.coviro.2014.02.007 ; PMCID: PMC4028408 24636947 PMC4028408

[7] Buchmeier M, de la Torre J, Peters C. Arenaviridae: the viruses and their replication. Field’s virology. 5. Philadelphia, PA, USA: Wolter Kluwer Lippincott Williams & Wilkins; 2007. p. 1791–827.

[8] Cajimat MNB, Milazzo ML, Rollin PE, Nichol ST, Bowen MD, Ksiazek TG, et al. Genetic diversity among Bolivian arenaviruses. Virus Res. 2009;140(1–2):24–31. doi: 10.1016/j.virusres.2008.10.016 19041349 PMC2795640

[9] Aguilar PV, Camargo W, Vargas J, Guevara C, Roca Y, Felices V, et al. Reemergence of Bolivian hemorrhagic fever, 2007–2008. 2009. Available from: http://www.cdc.gov/EID/content/15/9/1526.htm 10.3201/eid1509.090017 PMC2819859 19788833

[10] Johnson KM, Wiebenga NH, Mackenzie RB, Kuns ML, Tauraso NM, Shelokov A, et al. Virus isolations from human cases of hemorrhagic fever in Bolivia. Proc Soc Exp Biol Med. 1965;118:113–8. doi: 10.3181/00379727-118-29772 14254520

[11] Patterson M, Seregin A, Huang C, Kolokoltsova O, Smith J, Miller M, et al. Rescue of a recombinant Machupo virus from cloned cDNAs and in vivo characterization in interferon (αβ/γ) receptor double knockout mice. J Virol. 2014;88(4):1914–23. Epub 2013/11/27. doi: 10.1128/JVI.02925-13 ; PMCID: PMC3911560 24284323 PMC3911560

[12] Bradfute SB, Stuthman KS, Shurtleff AC, Bavari S. A STAT-1 knockout mouse model for Machupo virus pathogenesis. Virol J. 2011;8:300. Epub 2011/06/14. doi: 10.1186/1743-422X-8-300 ; PMCID: PMC3126776 21672221 PMC3126776

[13] Koma T, Huang C, Aronson JF, Walker AG, Miller M, Smith JN, et al. The ectodomain of glycoprotein from the Candid#1 vaccine strain of Junin virus rendered Machupo virus partially attenuated in mice lacking IFN-αβ/γ receptor. PLoS Negl Trop Dis. 2016;10(8):e0004969. Epub 2016/08/31. doi: 10.1371/journal.pntd.0004969 ; PMCID: PMC5006991 27580122 PMC5006991

[14] Golden JW, Beitzel B, Ladner JT, Mucker EM, Kwilas SA, Palacios G, et al. An attenuated Machupo virus with a disrupted L-segment intergenic region protects guinea pigs against lethal Guanarito virus infection. Sci Rep. 2017;7(1):4679. Epub 2017/07/05. doi: 10.1038/s41598-017-04889-x ; PMCID: PMC5498534 28680057 PMC5498534

[15] Golden JW, Maes P, Kwilas SA, Ballantyne J, Hooper JW. Glycoprotein-specific antibodies produced by DNA vaccination protect guinea pigs from Lethal Argentine and Venezuelan hemorrhagic fever. J Virol. 2016;90(7):3515–29. Epub 2016/01/23. doi: 10.1128/JVI.02969-15 ; PMCID: PMC4794662 26792737 PMC4794662

[16] Bell TM, Bunton TE, Shaia CI, Raymond JW, Honnold SP, Donnelly GC, et al. Pathogenesis of Bolivian hemorrhagic fever in guinea pigs. Vet Pathol. 2016;53(1):190–9. Epub 2015/07/02. doi: 10.1177/0300985815588609 26139838

[17] Jain S, Shrivastava-Ranjan P, Flint M, Montgomery JM, Spiropoulou CF, Albariño CG. Development of reverse genetic tools to study Chapare and Machupo viruses. Virology. 2023;588:109888. Epub 2023/09/22. doi: 10.1016/j.virol.2023.109888 37774602 PMC11539271

[18] Patterson M. The development of a reverse genetics system for Machupo virus. University of Texas Medical Branch; 2014.

[19] Emonet SF, Seregin AV, Yun NE, Poussard AL, Walker AG, de la Torre JC, et al. Rescue from cloned cDNAs and in vivo characterization of recombinant pathogenic Romero and live-attenuated Candid #1 strains of Junin virus, the causative agent of Argentine hemorrhagic fever disease. J Virol. 2011;85(4):1473–83. Epub 2010/12/01. doi: 10.1128/JVI.02102-10 ; PMCID: PMC3028888 21123388 PMC3028888

[20] Sánchez AB, de la Torre JC. Rescue of the prototypic Arenavirus LCMV entirely from plasmid. Virology. 2006;350(2):370–80. doi: 10.1016/j.virol.2006.01.012 16476461

[21] Flatz L, Bergthaler A, de la Torre JC, Pinschewer DD. Recovery of an arenavirus entirely from RNA polymerase I/II-driven cDNA. Proc Natl Acad Sci U S A. 2006;103(12):4663–8. doi: 10.1073/pnas.0600652103 16537369 PMC1450228

[22] Koma T, Huang C, Coscia A, Hallam S, Manning JT, Maruyama J, et al. Glycoprotein N-linked glycans play a critical role in arenavirus pathogenicity. PLoS Pathog. 2021;17(3):e1009356. Epub 2021/03/01. doi: 10.1371/journal.ppat.1009356 ; PMCID: PMC7951981 33647064 PMC7951981

[23] Mantlo EK, Maruyama J, Manning JT, Wanninger TG, Huang C, Smith JN, et al. Machupo virus with mutations in the transmembrane domain and glycosylation sites of the glycoprotein is attenuated and immunogenic in animal models of Bolivian hemorrhagic fever. J Virol. 2022;96(8):e0020922. Epub 2022/03/28. doi: 10.1128/jvi.00209-22 ; PMCID: PMC9044957 35343792 PMC9044957

[24] Schmid NS, Clauss M, Hetzel U, Riond B, Bochmann M, Hatt J-M. Development, diagnosis and therapy of ketosis in non-gravid and non-lactating Guinea pigs. BMC Vet Res. 2020;16(1):41. doi: 10.1186/s12917-020-2257-2 32013972 PMC6998326

[25] Bell TM, Shaia CI, Bunton TE, Robinson CG, Wilkinson ER, Hensley LE, et al. Pathology of experimental Machupo virus infection, Chicava strain, in cynomolgus macaques (Macaca fascicularis) by intramuscular and aerosol exposure. Vet Pathol. 2015;52(1):26–37. Epub 2014/07/02. doi: 10.1177/0300985814540544 24990481

[26] Jahrling PB, Peters CJ. Passive antibody therapy of Lassa fever in cynomolgus monkeys: importance of neutralizing antibody and Lassa virus strain. Infect Immun. 1984;44(2):528–33. doi: 10.1128/iai.44.2.528-533.1984 ; PMCID: PMC263556 6715049 PMC263556

[27] Parekh BS, Buchmeier MJ. Proteins of lymphocytic choriomeningitis virus: antigenic topography of the viral glycoproteins. Virology. 1986;153(2):168–78. doi: 10.1016/0042-6822(86)90020-6 2426862

[28] Sommerstein R, Flatz L, Remy MM, Malinge P, Magistrelli G, Fischer N, et al. Arenavirus glycan shield promotes neutralizing antibody evasion and protracted infection. PLoS Pathog. 2015;11(11):e1005276. Epub 2015/11/20. doi: 10.1371/journal.ppat.1005276 ; PMCID: PMC4654586 26587982 PMC4654586

[29] Watanabe Y, Raghwani J, Allen JD, Seabright GE, Li S, Moser F, et al. Structure of the Lassa virus glycan shield provides a model for immunological resistance. Proc Natl Acad Sci U S A. 2018;115(28):7320–5. Epub 2018/06/25. doi: 10.1073/pnas.1803990115 ; PMCID: PMC6048489 29941589 PMC6048489

[30] Bonhomme CJ, Capul AA, Lauron EJ, Bederka LH, Knopp KA, Buchmeier MJ. Glycosylation modulates arenavirus glycoprotein expression and function. Virology. 2011;409(2):223–33. Epub 2010/11/05. doi: 10.1016/j.virol.2010.10.011 ; PMCID: PMC3053032 21056893 PMC3053032

[31] Stanfield RL, Berndsen ZT, Huang R, Sok D, Warner G, Torres JL, et al. Structural basis of broad HIV neutralization by a vaccine-induced cow antibody. Sci Adv. 2020;6(22):eaba0468. doi: 10.1126/sciadv.aba0468 32518821 PMC7253169

