# Supplementary material for: Development and characterization of a reverse genetics system for the lineage II Chicava strain of Machupo virus in a guinea pig model
Source: PLoS Negl Trop Dis. 2025 Jan 24;19(1):e0012834. doi: 10.1371/journal.pntd.0012834 (PMC11778707; doi:10.1371/journal.pntd.0012834)
Supplement: S1 Table — (DOCX) [file pntd.0012834.s002.docx]

**Supplementary Table 1. Amino acid differences in the NP and L protein between the Carvallo and Chicava strains**

| Viral Protein | Domains |  |
| --- | --- | --- |
| NP | N-terminal | F322L |
|  | Linker | - |
|  | C-terminal | I473V, ***Y499H****, ***G511E****, S548A |
|  |  |  |
| L | PA-like | E18D, G23A, I160M, ***G166E****, L244M, Q302S, V309A, S320T, D366E, V400I, H430L, H431Q, S433N, K437R, H450Y, T652I, S680G |
|  | RdRp | M718I, D863E, T886S, A896T, H910Y, T928I, P977S, ***P978D****, G1000E, V1020M, D1021E, N1277S, I1335V, A1401S, T1437A, E1554D, H1486Y |
|  | PB2-like | V1647I, S1648N, A1649V, T1651I, N1666Q, T1753A, ***Q1927K****, I1933V, ***E1935V****, K1964R, I1976T, E1981D, S2012T, V2014I, ***R2047S****, F2099L, S2116G, ***K2151I****, S2201N, P2203L |

* indicates non-conservative changes.
