# Supplementary material for: Development and characterization of a reverse genetics system for the lineage II Chicava strain of Machupo virus in a guinea pig model
Source: PLoS Negl Trop Dis. 2025 Jan 24;19(1):e0012834. doi: 10.1371/journal.pntd.0012834 (PMC11778707; doi:10.1371/journal.pntd.0012834)
Supplement: S2 Table — (DOCX) [file pntd.0012834.s003.docx]

**Supplementary Table 2.** Serum PRNT_50_ titers for animals challenged with respective MACV strains. Asterisks indicate surviving animals.

| **Animal group /tag number** | **Timepoint for collection (dpi)** | **PRNT_50_ titer (wtMACV Carvallo)** | **PRNT_50_ titer (rMACV Carvallo GPC_ΔN83/N166/F438I_)** |
| --- | --- | --- | --- |
|  |  |  |  |
| wtMACV Carvallo |  |  |  |
| #31 | 18 | <30 | 30 |
| #32 | 20 | <30 | 30 |
| #33* | 30 | <30 | 240 |
| #34 | 19 | <30 | 240 |
| #35 | 20 | <30 | 30 |
|  |  |  |  |
| rMACV Carvallo |  |  |  |
| #36 | 17 | <30 | 60 |
| #37 | 18 | <30 | <30 |
| #38* | 30 | 60 | 480 |
| #39* | 30 | 120 | 480 |
| #40* | 30 | 60 | 120 |
|  |  |  |  |
| wtMACV Chicava |  |  |  |
| #42 | 19 | <30 | 60 |
| #43 | 19 | <30 | 60 |
| #44 | 20 | <30 | 30 |
| #45 | 20 | <30 | 60 |
|  |  |  |  |
| rMACV Chicava |  |  |  |
| #46 | 20 | <30 | 60 |
| #47 | 19 | <30 | <30 |
| #48 | 21 | <30 | 30 |
| #49* | 30 | <30 | 120 |
| #50 | 19 | <30 | <30 |
|  |  |  |  |
